# Chemical composition and *in vitro* digestibility of corn stover during field exposure and the fermentation characteristics of silage prepared with microbial additives

**DOI:** 10.5713/ajas.18.0886

**Published:** 2019-04-15

**Authors:** Jun Lei Gao, Peng Wang, Chang Hai Zhou, Ping Li, Hong Yu Tang, Jia Bao Zhang, Yimin Cai

**Affiliations:** 1 College of Animal Science, Jilin University, Changchun 130062, China; 2 School of Architecture and Civil Engineering, Changchun Sci-Tech University, Changchun 130600, China; 3 Japan International Research Center for Agricultural Sciences, Tsukuba 305-8686, Japan

**Keywords:** Cellulase, Corn Stover, *In vitro* Digestibility, Lactic Acid Bacteria, Silage Fermentation

## Abstract

**Objective:**

To effectively use corn stover resources as animal feed, we explored the chemical composition and *in vitro* digestibility of corn stover during field exposure and the fermentation characteristics of silage prepared with lactic acid bacteria (LAB) and cellulase.

**Methods:**

Corn ears including the cobs and shucks were harvested at the ripe stage. The corn stover was exposed in the field under natural weather conditions. Silages were prepared after 0, 2, 4, 7, 15, 30, and 60 d of exposure. Corn stover was chopped into approximately 1 to 2 cm lengths and then packed into 5 liter plastic silos. The ensiling density was 550.1±20.0 g/L of fresh matter, and the silos were kept at room temperature (10°C to 25°C). Silage treatments were designed as follows: without additives (control), with LAB, with cellulase, and with LAB+ cellulase. After 45 d of fermentation, the silos were opened for chemical composition, fermentation quality and *in vitro* digestion analyses.

**Results:**

After harvest, corn stover contained 78.19% moisture, 9.01% crude protein (CP) and 64.54% neutral detergent fiber (NDF) on a dry matter (DM) basis. During field exposure, the DM, NDF, and acid detergent fiber (ADF) contents of corn stover increased, whereas the CP and water-soluble carbohydrate contents and *in vitro* digestibility of the DM and CP decreased (p<0.05). Compared to the control silage, cellulase-treated silage had lower (p<0.05) NDF and ADF contents. The pH values were lower in silage treated with LAB, cellulase, or LAB+cellulase, and lactic acid contents were higher (p<0.05) than those of the control. Silage treated with cellulase or LAB+cellulase improved (p<0.05) the *in vitro* DM digestibility (IVDMD) compared to that of the control or LAB-treated silage.

**Conclusion:**

Corn stover silage should be prepared using fresh materials since stover nutrients are lost during field exposure, and LAB and cellulase can improve silage fermentation and IVDMD.

## INTRODUCTION

Crop byproducts from agriculture are an important animal feed resource worldwide. In China, approximately 900 million tons of crop straw are produced annually, of which 30% is corn stover. After harvest, corn stover generally remains fresh and contains nutrients suitable for animal feed. Currently, at least 30% of corn stover is used for animal production or papermaking, whereas the remainder is usually incinerated in the field or plowed back into the soil after pulverization [1]. In recent years, the continuous degeneration of native grasslands has created a structural shortage of feed resources for herbivorous animal husbandry. Therefore, the effective use of corn stover resources to preserve fermented feed has become an urgent research topic globally, particularly in China.

Ensilage is an effective method for processing and utilizing forage. Corn stover silage is currently widely used as ruminant feed in animal husbandry. However, following corn grain harvest, when exposure time is prolonged, corn stover moisture and nutrients decrease and lignification increases, resulting in decreased digestibility for animals [2]. Therefore, selecting the proper time for ensilage following corn harvest is an important condition for obtaining high-quality corn stover silage.

Microbial additives such as lactic acid bacteria (LAB) and cellulase are widely used in silage preparation [3]. LAB plays an important role in aiding fermentation and preventing spoilage. Cellulase acts as a biocatalytic agent in the decomposition of cellulose; as a water-soluble carbohydrate (WSC), the cellulose decomposition product can be used by LAB to promote fermentation [4]. Many studies have demonstrated that using LAB or cellulase additives improves fermentation quality and nutritive value [5].

Despite previous studies focusing on forage, grass, and whole-crop corn silage [5,6], very little information is available on fermentation of corn stover silage at different exposure stages. As a part of the research related to effective utilization of corn stover, our previous study using different cultivars of corn confirmed that with increasing field exposure of corn stover, the nutrient and *in vitro* digestibility decreased [7]. Whether microbe additives can improve silage fermentation and nutrition value of corn stover silage requires future study. In this study, we prepared corn stover silage during field exposure using LAB and cellulase and evaluated the characteristics of the resulting fermentation.

## MATERIALS AND METHODS

### Material and ensilage

The corn (*Zea mays* L.) that is widely cultivated in northeastern China was selected for use in this experiment. The cultivation experiment was carried out in an experimental field (125.4°E, 43.9°N, Changchun, China). Seeds were sown on May 5, 2016, and weeds were removed by application of herbicide on May 25, 2016.

Corn ears including cobs and shucks were harvested at the ripe stage on September 26, 2016. The corn stover was exposed in the field under natural weather conditions. During the experiment, the average temperature was 3°C, and the average air humidity was 71.3%. Silages were prepared after 0, 2, 4, 7, 15, 30, and 60 d of exposure. The corn stover with 0, 2, 4, and 7 d of exposure was prepared directly, and the corn stover exposed for 15, 30, and 60 d was adjusted to 60% moisture for producing silage. The weighed LAB, cellulose, and LAB+cellulase were dissolved in 20 mL of distilled water, uniformly sprayed on corn stover in a pot and mixed well.

Silage treatments were designed as follows: without additives (control); with LAB inoculant Chikusou-1 (LAB, *Lactobacillus plantarum*, Snow Brand Seed Co., Ltd, Sapporo, Japan); with *Acremonium cellulase* enzyme (cellulase, Meiji Seika Pharma Co., Lte, Tokyo, Japan); or with LAB+cellulase. The LAB was inoculated at 1.0×10^5^ colony-forming units/g of fresh matter (FM), and cellulase was applied at 50 mg/kg of FM.

Corn stover was chopped into approximately 1 to 2 cm lengths before ensiling and then packed into 5 liters plastic silos. The ensiling density was 550.1±20.0 g/L of FM, and the silos were kept at room temperature (10°C to 25°C). After 45 d of fermentation, three replicate silos were opened for chemical composition, fermentation quality and *in vitro* digestion analyses.

### Chemical analysis

Dry matter (DM) of fresh samples and silage were determined by oven drying at 65°C for 48 h. Then, these oven-dried samples were milled through a 1.0 mm screen for chemical analyses. Organic matter (OM), crude protein (CP), and ether extract (EE) were analyzed according to the methods of the Association of Official Analytical Chemists [8]. Neutral detergent fiber (NDF), acid detergent fiber (ADF) and acid detergent lignin (ADL) were determined according to Van Soest et al [9]. WSC was determined using the sulfuric acid-anthrone method [10]. Buffering capacity (BC) was measured by the method of Playne and McDonald [11]. Gross energy (GE) was determined by oxygen bomb calorimeter [8]. Digestive energy (DE), metabolizable energy (ME), net energy for maintenance (NEm), net energy for lactating cows (NEl), and net energy for gain (NEg) were calculated using the following formulas.

DE=GE×[70.19-1.364×(ADF-29.83)-3.94+0.104×CP+0.149×EE+0.022×NDF-0.244×ash]/100

This formula was based on 347 experimental data from France and other countries, some of which came from castrated rams fed separately from hay and a mixture of hay and concentrate and some from studies that fed 50,070 dairy cows different ratios of fine and coarse mixed diets. The crude fiber (CF) content in the feed varied from 130 to 410 g/kg (mean 243 g/kg), and the total protein content was 86 to 330 g/kg (mean 166 g/kg). This formula has been accurately verified by the results of trials with castrated rams fed 17 different diets. The following formulas were also derived from the above formula [12].

$$\begin{array}{l} \text{ME}=\text{DE}\times [86.38-(9.9\times \text{CF}+19.6\times \text{CP})/(100-\text{ash})]/100\\ \text{NEm}=\text{ME}\times (0.287\times \text{ME}/\text{GE}+0.554)\\ \text{NEl}=\text{ME}\times (0.24\times \text{ME}/\text{GE}+0.463)\\ \text{NEg}=\text{ME}\times (0.78\times \text{ME}/\text{GE}+0.006) \end{array}$$

Herein, except for CF (g/kg), CP, EE, NDF, ADF, and Ash are expressed as a percentage of DM (% DM); the unit of DE, ME, NEm, NEl, and NEg is kJ/g of DM.

### Fermentation analysis

Twenty grams of each silage sample were homogenized in a blender with 180 mL of distilled water for 1 min and then filtered through four layers of cheesecloth as described by Owens et al [13]. The filtrate was used to measure pH (PHSJ-4F; INESA Co. Ltd., Shanghai, China), ammonia nitrogen (ammonia-N) and organic acid contents. The ammonia-N concentration was determined by Robinson [14]. The organic acid contents, including lactic acid (LA), acetic acid (AA), propionic acid (PA), and butyric acid (BA), were determined by high-performance liquid chromatography (column: Shodex RS Pak KC-811; Showa Denko K. K., Kawasaki, Japan; detector: DAD, 210 nm, SPD-20A; Shimadzu Co., Ltd, Kyoto Japan; eluent: 3 mmol/L HCLO_4_, 1.0 mL/min; temperature: 50°C).

### *In vitro* digestibility

The *in vitro* DM digestibility (IVDMD) was measured (0.5 g DM per sample) by a two-step approach [15] using ruminal liquor from sheep fed alfalfa hay and whole-plant corn silage once a day. Rumen fluid was collected through the rumen cannulas 2 h after feeding and diverted to plastic bottles. The fluid was filtered through 4 layers of cheesecloth and combined on an equal volume basis. The combined filtrate was mixed with CO_2_-bubbled McDougall’s artificial saliva at a ratio of 1:4 (vol/vol), and the pH of the artificial saliva was 6.8. Then, 50 mL of buffered rumen fluid was transferred to 128 mL serum bottles containing 0.5 g of sample and flushed with O_2_-free CO_2_. Tubes were capped with a butyl rubber stopper and sealed with an aluminum cap. Incubations were performed at 39°C for 6 h in a water bath with a reciprocal shaker (100 strokes/min). Then, after this procedure, measuring the concentrations of OM, CP, and GE [8] in the indigestible residue left in the test tubes allowed the estimation of the *in vitro* OM digestibility (IVOMD), *in vitro* CP digestibility (IVCPD), and *in vitro* GE digestibility (IVGED) [16].

### Animal care

Animal experiments were approved by the Committee of Animal Experimentation and were performed under the institutional guidelines for animal experiments of the College of Animal Science, Jilin University, China. The experiments were performed according to recommendations proposed by the European Commission to minimize the suffering of animals.

### Statistical analysis

All data from this experiment were subjected to analysis with SAS ver. 9.1 (SAS Institute, Cary, NC, USA). For the chemical compositions, BC, energy, and *in vitro* digestibility of corn stover, the significance differences among means were first analyzed using one-way analysis of variance (ANOVA), followed by Duncan’s test for significance between means. Linear, quadratic, cubic, logarithmic, monomial, and exponential regression models were fitted to describe the relationship between days of exposure and measurements. All the regression models for all measurements were shown to be extremely significant (p<0.005), so the p value was not presented for each model in Table 1, 2. Data on the chemical composition, energy, fermentation quality, and *in vitro* digestibility of silage were analyzed using 3-factor ANOVA, with exposure d (D), additive LAB (A), and additive cellulase (B) as fixed factors. The main effect of each factor and the interactions between factors were analyzed. While the interaction between A and B was significant, levels of A and B were combined to a new factor (A+B) with 4 levels: no additives, LAB added, cellulase added, and both LAB+cellulase added. Then, the two-way ANOVA was used to test the main effects and interactions of exposure d (D) and AB. The contrasts between the main effects of marginal means were made with the least significant difference method.

## RESULTS

The chemical composition, BC, energy, and *in vitro* digestibility of corn stover, and the coefficient of determination for each regression equation (R^2^) during field exposure are shown in Table 1, 2. The DM, CP, NDF, and WSC values of fresh corn stover at 0 d of exposure were 21.91%, 9.01%, 64.54%, and 8.42% of DM, respectively. As the field exposure time increased, the DM, OM, NDF, ADF, and ADL contents significantly (p<0.05) increased in corn stover; however, the CP, WSC, DE, ME, NEm, NEl, NEg, and BC levels significantly (p<0.05) decreased. At 60 d of exposure, the CP, WSC, and DE decreased to 5.03, 3.41, and 7.98 MJ/kg of DM, respectively. As the field exposure time increased, the IVDMD, IVOMD, IVCPD, and IVGED of corn stover decreased significantly (p<0.05).

The chemical composition and energy of corn stover silage prepared with LAB and cellulase are shown in Table 3, 4. As the field exposure time increased, the CP content and GE, DE, ME, NEm, NEl, and NEg levels significantly (p<0.05) decreased, while NDF, ADF, and ADL contents significantly (p< 0.05) increased. Exposure d (D) influenced all chemical composition and energy measurements (p = 0.000), and LAB treatment of silage influenced the OM (p = 0.025). Cellulase treatment of silage influenced the NDF, ADF, DE, ME, NEm, NEl, and NEg (p = 0.002–0.008). D×LAB influenced the DM, OM, and CP (p = 0.000–0.002). D×cellulase influenced DM and GE (p = 0.000 and 0.001). LAB×cellulase influenced DM and GE (p = 0.005 and 0.002). D×LAB×cellulase influenced DM, OM, and CP (p = 0.000–0.009).

The fermentation characteristics of corn stover silage pre pared with LAB and cellulase are shown in Table 5. Silages prepared under all treatments were well preserved, with pH values below 4.10. As the field exposure time increased, corn stover silage pH increased significantly (p<0.05), but PA content decreased significantly (p<0.05). The LA and AA contents of corn stover silage were significantly (p<0.05) higher at 0 to 4 d of exposure than at 7 to 60 d of exposure. In LAB, cellulase or LAB+cellulase-treated silages, the pH was lower (p< 0.05), and LA and AA contents were higher (p<0.05) than those of the control, whereas the rates of ammonia-N/total N were not significantly different among treatments. D influenced all fermentation quality parameters (p = 0.000), and LAB treatment influenced LA and AA (p = 0.000). Cellulase treatment influenced pH, LA, AA, and BA (p = 0.000–0.004). D×LAB influenced LA, AA, and BA contents and ammonia-N/TN (p = 0.000). D×cellulase influenced pH and AA (p = 0.000). LAB×cellulase influenced DM and GE (p = 0.005 and 0.002). D×LAB×cellulase influenced LA, AA, PA, and ammonia-N/TN (p = 0.000–0.002).

The *in vitro* digestibility of corn stover silage prepared with LAB and cellulase is shown in Table 6. As the field exposure time increased, the IVDMD, IVOMD, IVCPD, and IVGED decreased (p<0.05). The IVDMD and IVCPD levels were higher (p<0.05) in cellulase- and LAB+cellulase-treated silages than in the control and LAB-treated silage. D and cellulase treatment influenced all measures *in vitro* digestibility (p = 0.000–0.003). D×LAB and D×LAB×cellulase influenced IVCPD (p = 0.004 and 0.000), but LAB and other interactions did not.

## DISCUSSION

Generally, as field exposure time increased, corn stover experienced moisture loss, whereas the proportion of DM increased significantly, and NDF, ADF, and ADL contents increased drastically [17]. These results are consistent with those of our study. We also demonstrated higher levels of NDF, ADF, and ADL components in corn stover, which further resulted in decreased IVDMD and DE, which were negatively correlated with cell wall structural components such as cellulose and lignin.

The WSC and CP content in corn stover also decreased significantly with exposure time (Table 1) due to the loss of feed nutrients, including noncellulosic saccharides. Aerobic spoilage bacteria present in the stover and plant respiration consume nutrients such as proteins and sugars in plants [18]; these processes are important factors contributing to the reduction in DE observed in this study. Sun et al [7] reported that fresh corn stover contained a relatively high LAB count and WSC content, and the resulting silage fermented well, with minimal nutrient loss and improved *in vitro* digestibility. Usually, fresh stover had a relatively low DM while high WSC content and LAB counts, result in the silage prepared as good quality, but the dry stover did not [7]. In agreement with the previous studies, with increasing field exposure of corn stover, the CP and WSC contents and *in vitro* digestibility decreased. Therefore, fresh corn stover has suitable ensiling characteristics, and that silage should be prepared immediately after harvesting corn.

Generally, as grasses grow to maturity, DM, NDF, ADF, and ADL levels increase, whereas WSC, CP, and EE levels decrease [19]. Silage material is typically required to contain at least 5% WSC DM for high-quality fermentation. Harvesting and exposure time greatly influence the chemical composition of forage; both WSC and BC contents tended to vary considerably [20], indicating that exposure time may change the chemical composition of the materials and influence silage fermentation quality.

In the current study, the NDF and ADF content was significantly lower in cellulase-treated silage than in control silage because the applied cellulase mainly degraded plant fiber (NDF and ADF) to increase WSC as a substrate for LAB to produce LA and improve silage fermentation [21]. The chemical composition did not differ significantly between silage treated with and without LAB and was similar across treatments at the same exposure time. It is likely that forage corn, including stover, has good ensilage characteristics, such that good silage fermentation preserves nutrients well.

Factors used to assess fermentation quality include the physiological properties of epiphytic LAB and fermentation products. Epiphytic LAB can reduce pH by synthesizing LA, inhibiting the activity of bacteria, fungi or plant enzymes, and decreasing microbial diversity, resulting in improved silage fermentation without the addition of LAB. Thus, good-quality silage fermentation slows the reaction process of protein degradation to form nonprotein nitrogen, ammonia nitrogen and volatile fatty acids, greatly preserving the CP and EE during ensilage [22]. Zhang et al [23] also found that in the anaerobic environment of natural fermentation, the conversion of WSC to LA allowed epiphytic LAB to lower the pH, which is consistent with the results of this experiment.

In cellulase-treated silage, ME and NEm levels were higher than those of the control silage, whereas NDF and ADF contents and the levels of other factors were lower (Table 3). This result demonstrates that cellulase could both reduce indigestible cellulosic materials and improve feed digestion and metabolism. It is worth noting that it could not be used alone in actual production because the CP and effective energy value of corn stover silage were low. Therefore, high-protein and high-energy feeds such as soybean meal, corn or high-quality hay should be used in combination to meet the demand for protein, NEl, and NEg in lactating cows or growing cattle when feeding cows or beef cattle with corn stover silage. In our study, D affected all chemical constituents of corn stover silage; LAB×cellulase did not affect the OM. D×LAB and cellulase treatment affected OM and GE levels. This result suggests that exposure time and the application of additives have a significant impact on material chemical composition and silage fermentation quality.

In all LAB and cellulase-treated silages, the pH was below 4.20 (Table 5), indicating high silage fermentation quality. As the exposure time increased, the LA content gradually decreased due to decreases in moisture and WSC content. However, the PA content of the LAB- or cellulase-treated silage was significantly higher than that of the control silage. The reason for this finding remains unclear; it may be that epiphytic propionibacteria in contact with stover material converted LA to PA and CO2 during ensilage. Future studies should aim to isolate and identify PA-producing bacteria.

The LA and AA content in corn stover silage was significantly higher at 0 to 2 d (Table 5) because the WSC of the silage material was higher at 0 to 2 d of exposure (7.33% to 8.42% DM); WSC was the main substrate for LAB fermentation. Jahanzad et al [24] found that molasses, a source of WSC, can increase the activity of homofermentative LAB and convert WSC to LA.

In the present study, the LA content of LAB+cellulase- treated silage was significantly higher than that of other silage treatments due to the conversion of cellulosic material into monosaccharides by cellulase, which enhanced the growth of LAB. However, at different exposure times, silage treated with LAB, cellulase and LAB+cellulase exhibited no significant differences from the control silage in ammonia-N/TN. This result could be explained by a high abundance of LAB, which can enhance fermentation quality, and by low pH, which inhibits the growth and proteolytic activity of clostridia [25]. During ensilage, clostridia cannot degrade protein to produce ammonia-N; therefore, it is impossible to obtain large quantities of ammonia-N. In this study, exposure time, additives and their interaction affected the pH and organic acid content but did not affect the ammonia-N/TN of silage. Thus, exposure time and additives can influence silage fermentation quality.

High fiber content generally leads to a decline in feed IVD MD [26]. In this study, IVDMD, IVOMD, IVCPD, and IVGED levels of corn stover silage were lower than those of unfermented corn stover due to a significant increase in NDF, ADF, and ADL contents (Table 6). Santos et al [27] found that after fermentation, IVDMD decreased by an average of 21.3% in three sugarcane shoots. Therefore, IVDMD was higher in the cellulase treatment than in the control and LAB treatments. OM digestibility is a key factor reflecting the nutritional intake efficiency and production value potential of animal feed; lignin content is negatively correlated with IVOMD [28] and is considered to be a major factor affecting plant cell wall digestibility [29]. In this study, corn stalk IVDMD and IVOMD decreased significantly as exposure time increased; the corn maturity, stem-to-leaf ratio, and the degree of stover lignification also increased, leading to reduced *in vitro* digestibility. This result was consistent with that of corn stover silage in our study. As the exposure time was prolonged, the CP content and energy decreased. Rain, sunlight and microbial growth during field exposure may contribute to declines in IVCPD and IVGED.

Exposure time and cellulase affected the *in vitro* digestibility of corn stover silage; however, the interaction between exposure time and LAB treatment had a significant impact only on IVCPD. Silage fermentation quality depends on the type of microorganisms applied; their growth is affected by two main factors: the exposure time of the microorganisms to the straw and the fermentation additive. The LA production inhibits the effects of other microorganisms. The growth substrate of these microorganisms is mainly protein, such that D×LAB mainly affects IVCPD.

## CONCLUSION

In this study, we examined the chemical composition and *in vitro* digestibility of field-exposed corn stover and the fermentation characteristics of silage prepared using microbial additives. Fresh corn stover had levels of certain nutrients that are characteristic of good ensilage materials. Corn stover generally lost nutrients during exposure. Ensilage preserved nutrients well, and our findings indicate that silage should be prepared using fresh stover. The addition of LAB and cellulase improved fermentation and *in vitro* digestibility of corn stover silage.

## Figures and Tables

**Table 1:** Chemical composition and buffering capacity of corn stover during field exposure

Exposure d	DM (%)	OM	CP	NDF	ADF	ADL	WSC	BC (mE/kg of DM)

		----------------------------------------------------- % of DM -------------------------------------------------------						
0	21.91^g^	90.38^d^	9.01^a^	64.54^f^	35.92^f^	4.93^d^	8.42^a^	266.62^a^
2	29.73^f^	91.37^c^	7.02^b^	66.48^e^	40.13^e^	6.21^c^	7.33^b^	197.05^c^
4	31.12^e^	93.60^b^	7.25^b^	70.73^d^	42.86^d^	6.96^bc^	6.02^c^	228.47^b^
7	35.72^d^	94.99^a^	5.99^c^	70.73^d^	42.86^d^	6.05^cd^	4.73^d^	203.58^c^
15	47.13^c^	94.81^a^	5.67^d^	77.92^c^	48.75^c^	8.14^ab^	4.16^e^	167.36^d^
30	71.35^b^	95.39^a^	5.01^e^	80.18^b^	51.39^b^	8.04^ab^	3.41^f^	173.02^d^
60	88.20^a^	95.12^a^	5.03^e^	82.10^a^	53.32^a^	8.98^a^	3.41^f^	128.61^e^
SEM	0.14	0.31	0.08	0.54	0.39	0.38	0.02	4.19
Coefficient of determination (R^2^) of regression equation (p<0.005)								
Linear	0.9319	0.3652	0.5168	0.7342	0.7395	0.6890	0.5453	0.6922
Quadratic	0.9767	0.6857	0.7934	0.9498	0.9423	0.8181	0.8462	0.7574
Cubic	0.9869	0.8693	0.8911	0.9898	0.9856	0.9000	0.9557	0.8650
Logarithmic	0.7911	0.7792	0.8996	0.9544	0.9830	0.8992	0.9199	0.8628
Monomial	0.7199	0.7782	0.9266	0.9561	0.9826	0.8904	0.9417	0.8581
Exponential	0.9904	0.3631	0.5798	0.7136	0.6982	0.6237	0.6272	0.7833

Data are means of the three silage samples.

DM, dry matter; OM, organic matter; CP, crude protein; NDF, neutral detergent fiber; ADF, acid detergent fiber; ADL, acid detergent lignin; WSC, water soluble carbohydrate; BC, buffering capacity; SEM, standard error of mean.

a–g Means within columns with different superscript letters differ significantly from each other (p<0.05).

**Table 2:** Energy and *in vitro* digestibility of corn stover during field exposure

Exposure d	Energy (MJ/kg of DM)						*In vitro* digestibility (% of DM)			
	
	GE	DE	ME	NEm	NEl	NEg	IVDMD	IVOMD	IVCPD	IVGED
0	18.97^e^	11.92^a^	9.66^a^	6.76^a^	5.66^a^	3.90^a^	52.16^a^	56.69^a^	50.52^a^	55.07^a^
2	19.19^cd^	10.99^b^	8.91^b^	6.12^b^	5.12^b^	3.28^b^	49.74^b^	54.12^b^	47.72^b^	52.99^b^
4	19.07^de^	10.20^c^	8.25^c^	5.59^c^	4.68^c^	2.83^c^	47.83^c^	52.16^c^	47.27^c^	52.25^c^
7	19.29^c^	10.38^c^	8.44^c^	5.73^c^	4.79^c^	2.93^c^	48.39^c^	52.69^c^	46.15^d^	52.03^c^
15	19.82^b^	9.17^d^	7.43^d^	4.91^d^	4.11^d^	2.22^d^	45.49^d^	49.15^d^	44.43^e^	49.05^d^
30	20.02^a^	8.59^e^	6.95^e^	4.54^e^	3.80^e^	1.93^e^	44.21^e^	47.71^e^	43.21^f^	44.99^e^
60	19.86^b^	7.98^f^	6.46^f^	4.18^f^	3.50^f^	1.68^f^	42.78^f^	46.28^f^	42.76^g^	46.62^f^
SEM	0.04	0.09	0.08	0.06	0.05	0.05	0.23	0.26	0.11	0.23
Coefficient of determination (R^2^) of regression equation (p<0.005)										
Linear	0.5850	0.7586	0.7582	0.7409	0.7409	0.7020	0.7414	0.7442	0.6560	0.7471
Quadratic	0.9516	0.9257	0.9249	0.9194	0.9194	0.9067	0.9112	0.9260	0.8996	0.9088
Cubic	0.9580	0.9742	0.9720	0.9711	0.9711	0.9688	0.9668	0.9744	0.9674	0.9600
Logarithmic	0.8310	0.9850	0.9824	0.9828	0.9828	0.9805	0.9829	0.9808	0.9789	0.9711
Monomial	0.8326	0.9810	0.9784	0.9798	0.9798	0.9788	0.9830	0.9798	0.9825	0.9707
Exponential	0.5848	0.8034	0.8029	0.7945	0.7945	0.7947	0.7648	0.7663	0.6752	0.7660

Data are means of the three silage samples.

DM, dry matter; GE, gross energy; DE, digestible energy; ME, metabolizable energy; NEm, net energy for maintenance; NEl, net energy for lactating cow; NEg, net energy for gain; IVDMD, *in vitro* dry matter digestibility; IVOMD, *in vitro* organic matter digestibility; IVCPD, *in vitro* crude protein digestibility; IVGED, *in vitro* gross energy digestibility; SEM, standard error of mean.

a–g Means within columns with different superscript letters differ significantly from each other (p<0.05).

**Table 3:** Chemical compositions of corn stover silage prepared with lactic acid bacteria and cellulase

Items	DM %	OM	CP	NDF	ADF	ADL

		-------------------------------------------------- % of DM ---------------------------------------------------				
Exposure d means						
0	21.31^a^	90.35^a^	7.66^e^	67.62^a^	40.49^a^	6.02^a^
2	21.28^a^	90.24^a^	7.09^d^	70.50^b^	43.55^b^	6.75^b^
4	27.01^b^	91.57^b^	7.15^d^	72.47^c^	45.44^c^	7.41^c^
7	27.74^c^	92.83^c^	6.41^c^	74.19^d^	46.05^c^	7.21^bc^
15	34.81^e^	93.35^d^	6.05^b^	76.17^e^	47.32^d^	7.67^c^
30	34.41^d^	94.53^f^	5.31^a^	77.54^f^	50.17^e^	8.98^d^
60	36.28^f^	94.22^e^	5.49^a^	77.88^f^	50.12^e^	9.28^d^
SEM	0.14	0.06	0.12	0.46	0.40	0.17
Additives means						
Control	29.01^ab^	92.53^b^	6.35^a^	74.39^b^	46.72^b^	7.74
LAB	28.81^a^	92.44^ab^	6.23^a^	74.16^ab^	46.43^b^	7.54
Cellulase	28.83^a^	92.46^ab^	6.41^ab^	73.14^a^	45.45^a^	7.47
LAB+cellulase	29.25^b^	92.33^a^	6.63^b^	73.39^ab^	46.04^ab^	7.72
SEM	0.11	0.05	0.09	0.35	0.30	0.13
Significance of main effects and interactions						
Exposure d (D)	0.000	0.000	0.000	0.000	0.000	0.000
LAB	0.316	0.025	0.530	0.987	0.610	0.824
Cellulase	0.249	0.065	0.012	0.006	0.008	0.708
D×LAB	0.000	0.002	0.001	0.695	0.636	0.447
D×cellulase	0.000	0.043	0.818	0.956	0.970	0.191
LAB×cellulase	0.005	0.725	0.058	0.498	0.147	0.079
D×LAB×cellulase	0.000	0.000	0.009	0.045	0.015	0.191

Data are means of the three silage samples.

DM, dry matter; OM, organic matter; CP, crude protein; NDF, neutral detergent fiber; ADF, acid detergent fiber; ADL, acid detergent lignin; SEM, standard error of mean; LAB, lactic acid bacteria.

a–f Means within columns with different superscript letters differ significantly from each other (p<0.05).

**Table 4:** Energy of corn stover silage prepared with lactic acid bacteria and cellulase

Items	GE	DE	ME	NEm	NEl	NEg

	------------------------------------------------------------ MJ/kg of DM -----------------------------------------------------					
Exposure d means						
0	19.70^b^	11.24^d^	9.09^d^	6.24^d^	5.22^d^	3.33^d^
2	20.27^c^	10.79^c^	8.73^c^	5.92^c^	4.95^c^	2.99^c^
4	19.70^b^	9.94^b^	8.04^b^	5.40^b^	4.51^b^	2.61^b^
7	19.73^b^	9.81^b^	7.94^b^	5.31^b^	4.44^b^	2.54^b^
15	20.23^c^	9.79^b^	7.90^b^	5.27^b^	4.40^b^	2.46^b^
30	18.98^a^	8.30^a^	6.69^a^	4.39^a^	3.67^a^	1.88^a^
60	18.91^a^	8.26^a^	6.66^a^	4.36^a^	3.65^a^	1.87^a^
SEM	0.05	0.11	0.09	0.07	0.06	0.06
Additives means						
Control	19.68^b^	9.61^a^	7.76^a^	5.18^a^	4.33^a^	2.45^a^
LAB	19.54^a^	9.59^a^	7.76^a^	5.19^a^	4.34^a^	2.47^a^
Cellulase	19.63^b^	9.91^b^	8.01^b^	5.39^b^	4.51^b^	2.63^b^
LAB+cellulase	19.72^b^	9.81^ab^	7.93^ab^	5.32^ab^	4.44^ab^	2.55^ab^
SEM	0.04	0.08	0.07	0.05	0.04	0.04
Significance of main effects and interactions						
Exposure d (D)	0.000	0.000	0.000	0.000	0.000	0.000
LAB	0.502	0.488	0.520	0.521	0.521	0.527
Cellulase	0.079	0.002	0.002	0.002	0.002	0.004
D×LAB	0.027	0.624	0.708	0.718	0.718	0.725
D×cellulase	0.001	0.693	0.684	0.741	0.741	0.838
LAB×cellulase	0.002	0.596	0.527	0.430	0.430	0.253
D×LAB×cellulase	0.083	0.039	0.052	0.040	0.040	0.020

Data are means of the three silage samples.

DM, dry matter; GE, gross energy; DE, digestible energy; ME, metabolizable energy; NEm, net energy for maintenance; NEl, net energy for lactating cow; NEg, net energy for gain; SEM, standard error of mean; LAB, lactic acid bacteria.

a–d Means within columns with different superscript letters differ significantly from each other (p<0.05).

**Table 5:** Fermentation quality of corn stover silage prepared with lactic acid bacteria and cellulase

Items	pH	LA	AA	PA	BA	Ammonia-N/TN (%)

		---------------------------------------- % of DM -----------------------------------------				
Exposure d means						
0	3.59^a^	8.15^d^	9.76^e^	0.87^b^	0.18^e^	2.26^a^
2	3.59^a^	7.18^c^	6.68^c^	0.94^b^	0.16^d^	2.37^c^
4	3.99^c^	8.07^d^	8.21^d^	0.35^a^	0.12^a^	1.66^a^
7	4.00^c^	6.23^b^	5.47^b^	0.25^a^	0.17^de^	2.17^bc^
15	3.85^b^	5.96^b^	1.07^a^	0.13^a^	0.14^bc^	2.10^bc^
30	3.98^c^	3.59^a^	1.30^a^	0.18^a^	0.13^ab^	2.29^bc^
60	4.05^d^	3.41^a^	1.58^a^	0.19^a^	0.15^c^	2.06^b^
SEM	0.01	0.20	0.22	0.09	0.01	0.09
Additives means						
Control	3.91^c^	5.20^a^	3.66^a^	0.28^a^	0.16^b^	2.17^ab^
LAB	3.87^b^	6.19^b^	5.32^bc^	0.58^b^	0.16^b^	2.25^b^
Cellulase	3.84^a^	6.16^b^	4.87^b^	0.54^b^	0.14^a^	2.10^ab^
LAB+cellulase	3.84^a^	6.79^c^	5.62^c^	0.27^a^	0.15^b^	2.00^a^
SEM	0.01	0.15	0.17	0.06	0.00	0.07
Significance of main effects and interactions						
Exposure d (D)	0.000	0.000	0.000	0.000	0.000	0.000
LAB	0.012	0.000	0.000	0.851	0.065	0.889
Cellulase	0.000	0.000	0.000	0.683	0.004	0.022
D×LAB	0.195	0.000	0.000	0.236	0.000	0.000
D×cellulase	0.000	0.062	0.000	0.952	0.045	0.022
LAB×cellulase	0.052	0.229	0.009	0.000	0.015	0.223
D×LAB×cellulase	0.103	0.000	0.000	0.000	0.198	0.002

Data are means of the three silage samples.

LA, lactic acid; AA, acetic acid; PA, propionic acid; BA, butyric acid; TN, total nitrogen; DM, dry matter; SEM, standard error of mean; LAB, lactic acid bacteria.

a–e Means within columns with different superscript letters differ significantly from each other (p<0.05).

**Table 6:** *In vitro* digestibility of corn stover silage prepared with lactic acid bacteria and cellulase

Items	IVDMD	IVOMD	IVCPD	IVGED

	----------------------------------------------------------- % of DM ---------------------------------------------------------			
Exposure d means				
0	50.06^d^	53.89^d^	48.21^e^	52.63^d^
2	48.72^c^	51.94^c^	46.67^d^	50.85^c^
4	47.17^b^	50.84^b^	46.55^d^	50.11^b^
7	46.89^b^	50.66^b^	45.75^c^	50.16^b^
15	46.76^b^	50.13^b^	45.12^b^	49.83^b^
30	43.32^a^	47.77^a^	43.76^a^	47.70^a^
60	43.20^a^	47.69^a^	43.94^a^	47.56^a^
SEM	0.25	0.26	0.14	0.24
Additives means				
Control	46.27^a^	50.06^a^	45.54^a^	49.54^a^
LAB	46.25^a^	50.15^a^	45.51^a^	49.57^a^
Cellulase	47.02^b^	50.91^b^	45.89^b^	50.27^b^
LAB+cellulase	46.81^b^	50.54^ab^	45.94^b^	49.97^ab^
SEM	0.19	0.20	0.10	0.18
Significance of main effects and interactions				
Exposure d (D)	0.000	0.000	0.000	0.000
LAB	0.522	0.517	0.912	0.447
Cellulase	0.001	0.003	0.000	0.003
D×LAB	0.530	0.596	0.004	0.532
D×cellulase	0.693	0.940	0.957	0.912
LAB×cellulase	0.590	0.276	0.682	0.350
D×LAB×cellulase	0.023	0.018	0.000	0.023

Data are means of the three silage samples.

IVDMD, *in vitro* dry matter digestibility; IVOMD, *in vitro* organic matter digestibility; IVCPD, *in vitro* crude protein digestibility; IVGED, *in vitro* gross energy digestibility; DM, dry matter; SEM, standard error of mean; LAB, lactic acid bacteria.

a–e Means within columns with different superscript letters differ significantly from each other (p<0.05).
